# Anticholinesterase activities of cold and hot aqueous extracts of *F. racemosa* stem bark

**DOI:** 10.4103/0973-1296.62897

**Published:** 2010-05-05

**Authors:** Faiyaz Ahmed, Asna Urooj

**Affiliations:** *Department of Studies in Food Science and Nutrition, University of Mysore, Manasagangotri, Mysore - 570 006, India*

**Keywords:** Acetylcholinesterase, Ayurveda, Alzhiemer's, *Ficus racemosa*, medhya rasayana

## Abstract

The present study evaluated the anticholinesterase activity of cold and hot aqueous extracts of *Ficus racemosa* stem bark against rat brain acetylcholinesterase *in vitro*. Both the cold aqueous extract (FRC) and the hot aqueous extract (FRH) exhibited a dose dependent inhibition of rat brain acetylcholinesterase. FRH showed significantly higher (*P* ≤ 0.001) cholinesterase inhibitory activity compared to FRC; however, both the extracts did not show 50% inhibition of AChE at the doses tested (200-1000 μg ml^−1^). The IC_50_ values of 1813 and 1331 μg ml^−1^ were deduced for FRC and FRH, respectively (calculated by extrapolation using Boltzmann's dose response analysis).

## INTRODUCTION

Ayurveda, an alternative system of medicine in India, uses a number of plants for the treatment of a variety of diseases. The medhya rasayana are a group of medicines in Ayurveda known to act on the nervous system. These drugs mainly contain extracts from plants such as *Acorus calamus, jatamansi* and *Bacopa monnieri*. These medhya rasayana have been claimed to improve mental ability.[1] The acteylcholinesterase (AChE) is a biologically important enzyme that hydrolyzes acetylcholine (ACh), a neurotransmitter considered to play role in the pathology of Alzhiemer's disease.[2–3] One of the most important approaches for treatment of this disease involves the enhancement of acetylcholine level in brain using AChE inhibitors.[4] Several studies have reported anticholinesterase activity of the plant extracts and drugs.[5–8] Certain reports have claimed that, a few herbal extracts can act on the central nervous system, thereby enhancing the faculties of learning and memory. A recent study has shown that *B. monnieri* improves memory in humans.[9–10] *Clitoria ternatea* and *jatamansi* have also been reported to be excellent memory enhancers.[11]

*Ficus racemosa* Linn. (Moraceae) commonly known as ‘cluster fig’ found throughout greater part of India in moist localities is widely used in Indian folk medicine for the treatment of various diseases/disorders including jaundice, dysentery, diabetes, diarrhea and inflammatory conditions.[12] We have reported *F. racemosa* stem bark to possess excellent antioxidant properties *in vitro, ex vivo*[13] and *in vivo* in streptozotocin-induced diabetic rats,[14] antidiabetic activity *in vitro*[15] and antihyperglycemic activity *in vivo*,[16] hepatoprotective activity against CCl_4_ induced hepatotoxicity[17] and carbohydrate hydrolyzing enzyme inhibitory activity.[18] However, no reports are available on the anticholinesterase activity of *F. racemosa* bark, hence the present investigation was undertaken to evaluate the anticholinesterase activity of cold and hot water extracts of *F. racemosa* bark against rat brain AChE *in vitro*.

## MATERIALS AND METHODS

### Chemicals and plant material

Acetylthiocholine iodide and 5, 5-Dithio (bis) nitro benzoic acid (DTNB) were purchased from Sigma Aldrich, Bangalore, India. All the other reagents and chemicals used in the study were of extra pure analytical grade. *Ficus racemosa* stem bark was collected from Mukkadahally, Chamarajanagar district of Karnataka, India during September 2007, subsequently identified by Dr. Shivprasad Huded, JSS Ayurvedic Medical College, Mysore, and the voucher specimen (BOT-001/2008) was deposited at the herbarium of Department of Studies in Botany, University of Mysore, Mysore, India. The bark was cut into small pieces, dried (50°C) and powdered, passed through 60 mesh sieve (BS) and stored in an air tight container at 4°C till further use.

### Preparation of the extracts

Cold aqueous extract (FRC) was prepared by extracting powdered *F. racemosa* bark (FRB) with distilled water (1:8 w/v) at room temperature on a mechanical shaker for 24 h, filtered and freeze dried. Hot aqueous extract (FRH) was prepared by extracting FRB with distilled water (1:8 w/v) at 70°C in a temperature controlled mechanical shaker for 24 h, filtered and freeze dried (Yield; FRC: 9.5%, FRH: 12% w/v). Their percentages were calculated in terms of initial air dried plant material.[19]

### *In vitro* acteylcholinesterase inhibition assay

AChE inhibition activities of selected extracts were measured by slightly modifying the spectrophotometric method developed by Ellman.[^20^] Acetylthiocholine iodide was used as substrate and 5, 5 -Dithiobis [2-nitrobenzoic acid] was used for the measurement of cholinesterase activity and rat brain homogenate was used as source of acteylcholinesterase enzyme.

### Preparation of the enzyme

Male rat of Wistar strain weighing 150 g was sacrificed by cervical dislocation, the brain was immediately excised and homogenized with 0.1 mM sodium phosphate buffer (pH 7.0) in cold condition. The homogenate was stored at -80°C till use.

### Assay procedure

Various concentrations of the extracts in 2.6 ml of 0.1 mM sodium phosphate buffer (pH 8.0) were added to 100 μl of DTNB (0.75 mM) and 5 μl brain homogenate (Crude enzyme) and incubated for 5 min at 25°C. The reaction was then initiated by the addition of 20 μl of acetylthiocholine. The hydrolysis of acetylthiocholine was monitored by the formation of yellow 2-nitro-5-sulfidobenzene-carboxylate anion as the result of the reaction of DTNB with thiocholine, released by the enzymatic hydrolysis of acetylthiocholine for 10 min, at a wavelength of 412 nm. The percentage Inhibition of cholinesterase activity was calculated using the following formula.

$\text{Inhibition of AChE}\left(\%\right)=\frac{\Delta A\;control-\Delta A\;sample}{\Delta A\;control}\times 100$

Δ*A control* is the absorbance of the control reaction (containing all reagents except the test compound), and Δ*A sample* is the absorbance of the test compound. Neostigmine bromide was used as positive control and all tests were carried out in triplicate.

### Statistical analysis

Data was analyzed by ANOVA followed by Tukey's multiple comparisons test for significant differences using SPSS 14.0 software. IC_50_ values were calculated by Boltzmann's dose response analysis using Origin 6.1 software.

## RESULTS

**Figure 1:**
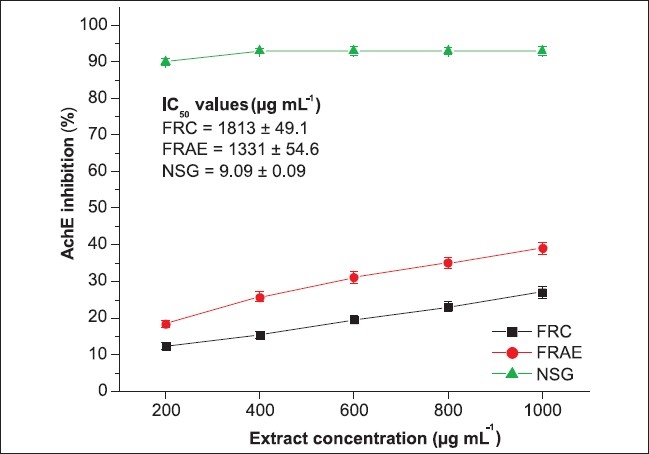
Anticholinesterase activity of FRC and FRH. *Values are mean ± SD of triplicate determinations; IC_50_ values were calculated by Boltzmann's dose response analysis using Origin 6.1 computer software; neostigmine bromide was used as positive control

The anticholinesterase activities of cold and hot aqueous extracts of the *F. racemosa* bark are presented here. Both the extracts (FRC and FRH) exhibited a dose dependent inhibition of rat brain acetylcholinesterase [Figure 1]. However, their inhibitory activities were significantly lower (*P* ≤ 0.001) than that of neostigmine bromide, a standard acetylcholinesterase inhibitor. Among FRC and FRH, FRH showed significantly higher (*P* ≤ 0.001) cholinesterase inhibitory activity compared to FRC; however, both the extracts did not show 50% inhibition of AChE at the doses tested (200-1000 μg ml-1) and hence IC50 values were calculated by extrapolation using Boltzmann's dose response analysis. On the basis of this analysis, IC_50_ values of 1813 and 1331 μg ml^−1^ were deduced for FRC and FRH, respectively, and the IC_50_ value of FRH was significantly lower (*P* ≤ 0.01) than that of FRC; however, these IC_50_ values were significantly lower (*P* ≤ 0.01) than the IC_50_ value of neostigmine.

## DISCUSSION

The present investigation reports the anticholinesterase activity of aqueous extracts of *F. racemosa* bark. AChE is found among neurofibrillary tangles and neuritic plaques[^21^] and its inhibition is an effective tool for the treatment of Alzheimer's disease and related Dementia.[^22^] Tacrine, a standard drug, exerts its pharmacological effect by increasing the acetylcholine level in the mouse brain.[^23^] All of the known acetylcholinesterase inhibiting drugs used in the therapy of AD suffers from several side effects such as high toxicity, short duration of biological action, low bioavailability and narrow therapeutic effects. Consequently, development of new acetylcholinesterase inhibitors with less toxicity and more potent activity is compulsory. Hence, the AChE inhibitory effects of plant extracts indicate their potential in the development of natural therapeutics for Alzheimer's disease and related problems.[^24^]

The search for new drugs, such as Huperzin A, with acetylcholinesterase inhibitory activity to be used in the treatment of AD from natural resources, also yielded some herbal-originated extracts and/or compounds such as *Ginkgo biloba, Panax ginseng, Davilla rugosa*, (-)-epigallocatechin, ferulic acid, etc. which act by different mechanisms.[^25^–^28^] However, acetylcholinesterase inhibitors have been accepted to be the most effective for the treatment of AD, to date. These observations indicate that, the available biodiversity of natural sources and the isolated bioactive compounds may act as potential leads for the development of clinically useful pharmaceuticals.[^29^]

In our study, although *F. racemosa* bark extracts did not inhibit AChE to a great extent, their inhibitory effects are of interest due to the fact that, *F. racemosa* is a rich source of phenolic compounds that are known to exhibit AChE-inhibitory activity. Although, alkaloids are considered to be the major anticholinesterase compounds found in plants,[^29^] recently we have reported a significant positive correlation between the total phenolic content and anticholinesterase activity of methanol extracts of *Acorus calamus* and *Nardostachys jatamansi*.[^24^]

## CONCLUSION

From the results of the present study, it is concluded that *F. racemosa* stem bark extracts possesses moderate anticholinesterase activity. There is a need to isolate and characterize the compounds responsible for the anticholinesterase activity for their effective utilization in the treatment of Alzheimer's disease and other stress related disorders. Studies in this direction are currently underway in our laboratory.
